# Frequency of Semen Collection Affects Ram Sperm Cryoresistance

**DOI:** 10.3390/ani12121492

**Published:** 2022-06-08

**Authors:** Cristina Palacin-Martinez, Mercedes Alvarez, Rafael Montes-Garrido, Marta Neila-Montero, Luis Anel-Lopez, Paulino de Paz, Luis Anel, Marta F. Riesco

**Affiliations:** 1 Assisted Reproduction Techniques Research Group (Itra-ULE), INDEGSAL, University of León, 24071 León, Spain; cpalm@unileon.es (C.P.-M.); rmong@unileon.es (R.M.-G.); mneim@unileon.es (M.N.-M.); lanel@unileon.es (L.A.-L.); ppazc@unileon.es (P.d.P.); laner@unileon.es (L.A.); mferrs@unileon.es (M.F.R.); 2 Animal Reproduction and Obstetrics, Department of Veterinary Medicine, Surgery and Anatomy, University of León, 24071 León, Spain; 3 Anatomy, Department of Veterinary Medicine, Surgery and Anatomy, University of León, 24071 León, Spain; 4 Cellular Biology, Department of Molecular Biology, University of León, 24071 León, Spain

**Keywords:** sperm cryopreservation, ram, sexual regime, GPX activity, SOD activity

## Abstract

**Simple Summary:**

Sperm cryopreservation is an important step to optimize artificial insemination protocols for rams. Traditionally, the improvement of ram sperm cryopreservation protocols has been addressed through different methods: formulation of different cryoprotectants, supplementation with antioxidants or carrying out different freezing-thawing rates. However, the influence of the male sexual regime as a factor for the improvement of freezability has not been studied until now. The frequency of sperm collection in the work protocols of reproduction centers can be variable depending on the dose demand. To corroborate the variability of the quality of thawed semen according to the frequency of collection, in addition to traditional analyses, new tests based on the redox balance of spermatozoa were performed. All these aspects have been applied and discussed extensively throughout this manuscript. In addition, new approaches, such as antioxidant activity of some enzymes have been incorporated to provide more precision in integrative studies of the redox state in spermatozoa. The deleterious effect triggered by a high dose demand should be demonstrated as a consequence of a redox imbalance.

**Abstract:**

The improvement of frozen-thawed sperm quality has been mostly approached from the view of cryopreservation protocol optimization in terms of cryoprotectant solutions, freezing-thawing rates and antioxidant supplementation, while the impact of sperm collection frequency remains unknown in rams. In this work, a multiparametric study was carried out in cooled and frozen-thawed semen to evaluate sperm quality after different semen collection frequencies during a month: zero sperm collection (0 CW), four sperm collections per week (4 CW), and ten sperm collections per week (10 CW). Traditional analyses have been applied, in combination with novel technologies related to redox balance. Frozen-thawed semen quality showed a significant decrease (*p* < 0.05) in 0 CW and 10 CW in comparison to 4 CW, concerning motility and kinetics parameters. However, apoptosis showed a significant increase (*p* < 0.05) in 10 CW in comparison to 0 CW and 4 CW. The employment methods related to redox balance provided us with the definitive probe to ensure the influence of collection frequency on balance redox after thawing. Specifically, glutathione peroxidase (GPX) and superoxide dismutase (SOD) activity showed a significant decrease (*p* < 0.05) in 10 CW compared to 0 CW and 4 CW. The characterization of alternative strategies to sperm cryopreservation based on consideration of male sexual regimes, could improve the quality of frozen-thawed sperm.

## 1. Introduction

Artificial insemination (AI) is a useful tool to improve productivity and high genetic traits [1]. In this sense, sperm cryopreservation is an important approach to optimize semen management used in AI technologies [2,3]. Cryopreservation makes the transport to different insemination centers, farms or other countries easier. Also, it allows sperm conservation for many years, which is important to preserve genetic variability in animal species, including ovine species [1,4,5]. Nevertheless, the use of frozen-thawed semen presents some constraints considering its performance decrease, especially affecting some sperm parameters, such as motility and viability [2,3,6]. It has been widely described that during the cryopreservation process lethal and sublethal freezing injuries in spermatozoa frequently appear [4]. Sperm are affected by temperature changes causing oxidative, chemic, osmotic and physic stress, ice glass formation, finally triggering low fertility rates [5]. Specifically, several sperm quality parameters affected by freezing have been described by different authors: motility and viability decrease [6,7], and lipid peroxidation [7] and reactive oxygen species (ROS) content increase [6] in frozen-thawed ram sperm [6,7,8]. In general terms, cryopreservation provokes the alteration of sperm redox homeostasis and this is one of the main causes of male infertility [9]. An alteration in the redox balance after thawing has been observed in the rest of the studies performed on mammalian sperm from different species including stallions [10] and humans [11]. Nevertheless, reactive oxygen species (ROS) are necessary to sperm physiology and to reach crucial functions (capacitation, maturation, acrosome reaction, or fertilization). Oxidative stress in sperm cells appears especially after freezing as a consequence of the temperature difference [7,12]. For this reason, sperm quality parameters related to redox balance should be included in cryopreservation studies. Traditionally, oxidative stress was evaluated by the measure of oxidant substances, such as malondialdehyde (MDA) levels in seminal plasma as a lipid peroxidation marker, it being a final product of polyunsaturated fatty acid peroxidation [13,14], total antioxidant capacity (TAC) [13,14] or ROS content using different fluorescence probes. An increase of ROS levels, and overall, an imbalance of this redox system, triggers lipid peroxidation, DNA fragmentation or apoptosis and consequently lowers fertility [9,14,15,16,17]. For this reason, redox studies should be multiparametric analyses, including several tests based on ROS concentration, specific antioxidants of the sample and redox balance. The analysis of the natural antioxidant capacity of sperm given by enzymatic antioxidants, such as superoxide dismutase (SOD), glutathione peroxidase (GPX), catalase (CAT), glutathione S-transferase (GST) and non-enzymatic antioxidants (glutathione, vitamin (A, C, E), have been largely employed to study redox status, due to its effect in counteracting excessive ROS in the sample [15,16]. In this sense, enzymatic antioxidants (SOD, GPX, CAT) play an important role in redox balance regulation, being responsible for maintenance of frozen sperm quality [17]. Specifically, SOD is the most antioxidant enzyme related to ROS detoxification in mammalian sperm [18,19] and has been considered as an important sperm quality marker [20]. SOD is a metalloenzyme that catalyzes the dismutation of the superoxide anion to molecular oxygen and hydrogen peroxide [21], while GPX catalyzes the reduction of hydroperoxides [22]. SOD and GPX activity were previously measured in ram seminal plasma: SOD and GPX levels were higher in rams supplemented with vitamin C and E by oral administration, in comparison to the control group (rams without oral supplementation) [23]. Different authors described low levels of these enzymatic antioxidants were related with high DNA damage in bulls [24] and low motility in stallions [25]. In recent years, different analyses related to oxidative stress have been described considering the beneficial and detrimental role of ROS and considering the redox balance as a more accurate parameter in oxidative stress studies [26]. The MiOXSYS^®^ system is a new technology to conjointly evaluate redox balance by a galvanostatic-method [27]. The MiOXSYS^®^ system allows detection of variations in oxidant capacity in semen samples [24]. To summarize, the analysis of oxidative stress should be addressed with different oxidative stress markers, such as enzymatic activity of antioxidants located in seminal plasma (SOD, GPX, CAT), and through considering redox balance in a global way.

Traditionally, the improvement of ram sperm cryopreservation protocols has been addressed through different strategies: formulation of different cryoprotectant solutions and extender mediums [28,29], supplementation with different compounds, such as antioxidants [30,31], or carrying out different freezing-thawing rates [32,33]. However, new emerging strategies could be applied in order to improve sperm cryopreservation in rams. In this sense, semen collection frequency (sexual regime) has been previously described as a factor that could influence sperm quality. Different authors have described negative effects after high semen collection frequency and after an abstinence period [34,35]. Specifically, there is some evidence that claims ram males subjected to an intensive extraction regime presented lower semen volume, concentration and progressive motility [36]. This decrease in sperm quality could trigger fertility consequences, considering some studies performed with boar where low fertility rates were registered when semen collection frequency increased [37]. Contrary to this, in the abstinence period, a rise in sperm viability and mass motility, accompanied by a lower acrosome alteration, were described in rams [38]. The influence of the semen collection regime on sperm quality has been demonstrated by different authors in some mammalian species, including rams [38] and stallions [39]. This factor could be employed as an emerging approach to improve sperm quality in frozen-thawed samples, considering that males with higher genetic value are usually the most used in insemination centers. The selection of a specific sperm collection regime for dose elaboration could be a feasible strategy to improve the quality of thawed sperm in this species.

The objective of this work is to investigate the effect of semen collection frequency on the quality of thawed sperm. For this purpose, we combined conventional analyses (motility and kinetic analyses), and multiparametric approaches based on flow cytometry (viability, apoptosis and ROS content), with innovative analyses like redox balance based on the MiOXSYS^®^ measure and SOD or GPX activity to obtain an in-depth vision of redox status of sperm after thawing. This evaluation could provide new strategies in AI centers concerning dose elaboration schedules, based on semen collection frequency, to improve sperm quality after thawing.

## 2. Materials and Methods

### 2.1. Animals

Sperm donors used for the experiments were twenty-one healthy adult Assaf rams (*Ovis aries*) aged between two and seven years. Rams were kept at the Animal Selection and Reproduction Centre of Junta de Castilla y León (CENSYRA) located in Villaquilambre (León), under a standard balanced diet. In addition, veterinary inspections were carried out to control reproductive and other aspects, as well as nutrition.

The study was executed following the Guidelines of the European Union Council (86/609/EU), modified by 2010/63/EU, according to the national laws (RD 2013) for the laboratory animals. The experimental instructions were approved by Animal Care and Use Committee at the University of León (Spain) (ÉTICA-ULE-013-2018).

### 2.2. Semen Collection and Sample Processing

Ejaculates were collected from trained males by artificial vagina method (40 °C) (IMV Tecnologies, L’Aigle, France). The semen collection was made during the breeding season, in three different increasing semen collection frequencies, based on the work protocols used in the reproduction centers, according to the dose demand: (i) zero semen collection per week (0 CW) during a period of a month without semen collection, (ii) after two semen collections days per week (with two ejaculates each collection day) for a period of a month, 4 semen collections per week (4 CW) and (iii) after a month of semen collections every day per week (from Monday to Friday) with two ejaculates each collection day, 10 semen collections per week (10 CW). Forty-two ejaculates (two per male) were obtained from each experimental group.

Following the routine working conditions of a reproduction center, the first and second ejaculates of each male of the same collection day were mixed, with the objective of homogenizing the sample. The ejaculates were evaluated immediately in terms of ejaculate volume (graduated tubes) and mass motility (5 μL drop in a microscope prepared with a warmed plate). Each ejaculate mixture was split into two subsamples to obtained two experimental groups of the same collection day: cooled and cryopreserved. Cooled and cryopreserved samples were diluted with the same volume (1:1) in INRA96 medium^®^ (IMV Tecnhologies, L’Aigle, France) and TES-TRIS-Fructose medium supplemented with 20% clarified egg yolk and 4% glycerol (TTFM) made by our group [32], respectively. Samples were immediately transported to the laboratory in a water bath at 37 °C. In the laboratory, sperm concentration was analyzed by a cell counter (Nucleocounter SP-100, ChemoMetec, Allerod, Denmark).

In cooled samples, semen was diluted down to 1600 × 10^6^ of spermatozoa/mL in INRA96 medium^®^, packed in 0.25 mL French straws and stored at 15 °C for 6 h, as a positive control, based on the dose elaboration of the reproduction centers. Samples were cryopreserved following the protocol previously described by our group [32,40]. Semen was diluted to 100 × 10^6^ sperm/mL in TTFM. Before that, the samples were refrigerated at −0.25 °C/min to 5 °C using a water bath in the refrigerated chamber. After 2 h of equilibration at 5 °C, the diluted samples were packed in 0.25 mL French straw. Then, using a programmable bio-freezer (Kryo 10 Series III; Planes PLC, Sunbury-on-Thames, UK) straws were frozen at a rate of −20 °C/min down to −100 °C and finally dropped in liquid nitrogen. Samples were kept in liquid nitrogen containers until thawing. Straws were thawed in a water bath at 65 °C for 5 s.

### 2.3. Sperm Motility and Kinetic Parameters

Cooled and frozen-thawed sperm samples of different experimental groups (0 CW, 4 CW, 10 CW) were evaluated using the CASA system (Computer Assisted Sperm Analysis) (Sperm Class Analyzer -SCA- 6.3.0.59; Microptic S.L., Barcelona, Spain) to determine sperm motility and kinetic parameters. Refrigerated samples were analyzed after 6 h of cooling, while the frozen-thawed samples were processed 10 min after thawing. One sample from each male was diluted to 2 × 10^7^ sperm/mL in TES-TRIS-Fructose medium supplemented with 1% egg yolk. The diluted sample was tempered at 37 °C on a warmed plate and immediately 5 μL were drooped into a Makler counting chamber (10 μm depth; Sepi Medical Instruments, Mumbai, India) and analyzed with SCA. Samples were analyzed with a 10× negative phase contrast objective in a Nikon Eclipse Microscope (Nikon, Tokyo, Japan), equipped with a Baster A312fc digital camera (Baster Vision Technologies, Ahrensburg, Germany). These parameters were evaluated to capture at 100 frames/s, using pre-defined SCA ram sperm settings, adapted by our group (particles with an area of 20–70 μm^2^). A total of 400 sperm were acquired in at least four different fields.

The kinetic parameters registered were: velocity according to the straight path (VSL, μm/s), linearity (LIN, %), amplitude of the lateral displacement of the sperm head (ALH, μm), total motility (TM), defined as the percentage of sperm with VCL > 15 μm/s, progressive motility (PM), defined as the percentage of sperm with VCL > 45 μm/s, and fast progressive motility (FPM), defined as the percentage of sperm with VCL > 75 μm/s.

### 2.4. Multiparametric Flow Cytometry Analyses

Cooled and frozen-thawed samples were processed to be analyzed by flow cytometry. Refrigerated samples were analyzed after 6 h at 15 °C, while frozen-thawed samples were processed 10 min after thawing. Different fluorochromes were combined to evaluate sperm quality: Zombie Violet™ (Biolegend, San Diego, CA, USA) was used to determine viability associated with membrane integrity; CellEvent™ Caspase-3/7 (Invitrogen, Eugene, OR, USA) acted as an apoptotic marker and CellROX™ Deep Red (Invitrogen, Eugene, OR, USA) as a marker of ROS content.

Each sample was diluted to 2 × 10^6^ sperm/mL in PBS solution (Merck, Madrid, Spain) and centrifuged for 15 s (MiniSpin Plus, Eppendorf, Hamburg, Germany). Then, the sperm pellet of each sample was incubated with 96 μL of Zombie Violet™ (1:1000 final dilution), 2 μL of CellEvent™ Caspase-3/7 (4 μM final concentration) and 2 μL of CellROX™ Deep Red (5 μM final concentration) in the dark at room temperature. After 30 min, another washing step was performed to stop the cell staining and the pellet was resuspended in 1 mL of PBS. Samples were analyzed in a flow cytometer (MACSQuant Analyzer 10, Miltenyi Biotech, Bergisch Gladbach, Germany), equipped with three lasers emitting at 405, 488, and 635 nm (violet, blue and red, respectively) and 10 photomultiplier tubes. Violet fluorescence was detected in V1 (excitation 405 nm, emission 450/50 nm), green fluorescence was detected in B1 (excitation 488 nm, emission 525/50 nm), and red fluorescence was detected in R1 (excitation 635 nm, emission 655–730 nm (655 LP + split 730). The system was controlled by MACS Quantify software (Miltenyi Biotech, Bergisch Gladbach, Germany). Data analysis was carried out by FlowJo v.10.2 (Ashland, Wilmington, DE, USA).

### 2.5. MiOXSYS^®^ Analysis

The oxidation-reduction potential was analyzed by measuring the electron transfer to an oxidant from a reductant substance. The MiOXSYS^®^ system (Luoxis Diagnostics, Inc., Englewood, CO, USA), galvanostat-based, measured each sample and consistently and evaluated sORP (the totally balance of oxidants and reductants in a sample, measured in millivolts (mV)) and cORP (quantity of oxidants store, reported in microcoulombs (μC)). Frozen-thawed samples were processed 10 min after thawing. Samples (1 × 10^6^ sperm) were washed with 100 μL of PBS solution and centrifuged for 15 s (MiniSpin Plus, Eppendorf, Hamburg, Germany). The supernatant was eliminated and the pellet was homogenized with 20 μL of PBS solution. Then, a 20 μL drop was applied to a sensor collocated in the MiOXSYS^®^ diagnostic system to start the analysis. Finally, the result was expressed in mV/10^6^ and μC/10^6^ for sORP and cORP, respectively. Ten frozen-thawed samples from the same males for each of the three experimental groups (thirty samples), were analyzed by MiOXSYS^®^.

### 2.6. SOD Assay

SOD enzyme activity was measured by the Superoxide Dismutase Assay Kit (Cayman Chemical Company, Ann Arbor, MI, USA). This commercial kit uses a tetrazolium salt for the detection of superoxide radicals generated by xanthine oxidase and hypoxanthine. Thawed samples were processed 10 min after thawing. Before starting, each sample was centrifuged at 10,000× *g* for 15 min at 4 °C and the seminal plasma was separated to be used. Solutions were prepared following the manufacturer´s instructions. Diluted seminal plasma (20 μL) was added to each well. Control tubes were provided by the manufacturer for constructing a standard curve. In addition, diluent replicates were included in the experiment to be considered as blank wells to avoid diluent interference on absorbance values. Reaction was initiated by Xanthine Oxidase and the plate was incubated for 30 min at room temperature in the dark. Absorbance was read at 440–460 nm using a plate reader (Biotek, Gene 5 Microplate Reader, Winooski, VT, USA). Finally, the SOD activity of the samples was calculated using the equation obtained from the linear regression of the standard curve, substituting the linearized rate for each sample. Ten frozen-thawed samples from the same males for each of the three experimental groups (thirty samples), were analyzed; duplicates of wells were carried out.

### 2.7. GPX Assay

GPX enzyme activity was measured by the Glutathione Peroxidase Assay Kit (Cayman Chemical Company, Ann Arbor, MI, USA). This commercial kit measures GPX activity indirectly by a coupled reaction with glutathione reductase (GR). Oxidized glutathione (GSSG), produced upon reduction of hydroperoxide by GPX, is recycled to its reduced state by GR and NADPH. Oxidation of NADPH to NADP+ is accompanied by a decrease in absorbance at 340 nm. Samples were processed 10 min after thawing. Before using the GPX kit, each sample was centrifuged at 10,000× *g* for 15 min at 4 °C and the seminal plasma was separated from the sperm pellet. Different solutions were prepared following the manufacturer’s instructions. The reaction was initiated by Cumene Hydroperoxide and the absorbance per minute was read at 430 nm using a plate reader (Biotek, Gene 5 Microplate Reader, Winooski, VT, USA). GPX enzyme activity of the samples were calculated using an equation. Ten frozen-thawed samples from the same males for each of the three experimental groups (thirty samples), with their well duplicates, were analyzed.

### 2.8. Statistical Analysis

Prism 8 (GraphPad Software, San Diego, CA, USA) and SPSSv.22 (SPSS Inc., Chicago, IL, USA) were used to represent the data and perform the statistical analysis. Significant differences were considered to have *p* values < 0.05. Data were submitted to Kolmogorov–Smirnov and Levene’s tests to verify the normality and homogeneity of variances, respectively. Data were analyzed by one-way ANOVA or Kruskal–Wallis in non-normally distributed data. Differential rate (DR) (cooling/freezing) was the difference between the values measured in the cooled vs. frozen-thawed samples. DR in percentage was calculated (|cryo∗100/cooled| in each parameter as a measure of the decrease in sperm quality caused by cryopreservation, avoiding the initial sperm quality differences (cooled sperm is considered as an initial value). Results were expressed as the mean ± S.E.M. Pearson and Spearman correlation coefficients were calculated between seminal parameters. The reliability of the scoring systems was tested with the correlation coefficient (R squared). A principal component analysis (PCA), including all sperm quality parameters in the three experimental groups (0 CW, 4 CW, 10 CW), was performed for the set of sperm quality markers. The number of asterisks (*) indicated the significance levels: one asterisk (*) indicated *p* < 0.05, two asterisks (**) indicated *p* < 0.01, three asterisk (***) indicated *p* < 0.001, four asterisks (****) indicated *p* < 0.0001.

## 3. Results

### 3.1. Sperm Motility and Kinetics Parameters

**Figure 1:**
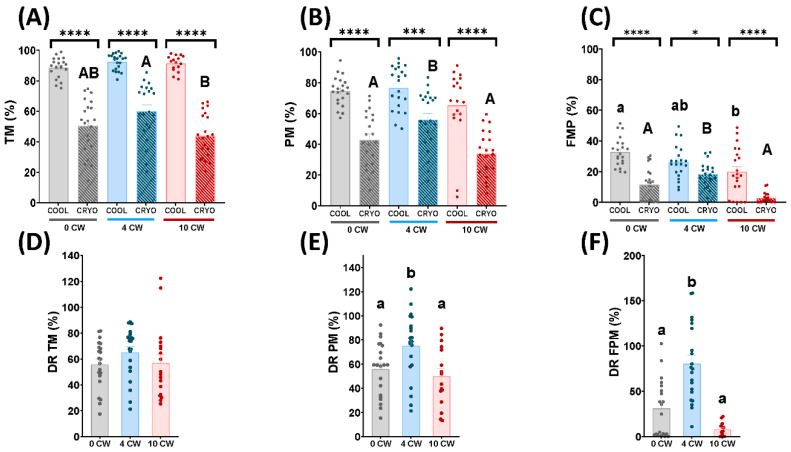
Ram sperm motility in cooling (COOL) and frozen-thawed (CRYO) samples and differential rate (cooling/freezing) depending on the sperm collection frequency: without sperm collection (0 CW), 4 sperm collections per week (4 CW), 10 sperm collections per week (10 CW). (**A**) Total motility (TM, %); (**B**) Progressive motility (PM, %); (**C**) Fast progressive motility (FPM, %) (**D**) Differential rate of total motility (TM, %); (**E**) Differential rate of progressive motility (PM, %); (**F**) Differential rate of fast progressive motility (FPM, %). Significant differences (*p* < 0.05) among experimental groups in cooling samples are represented with different lowercase superscripts letters (a, b). Significant differences (*p* < 0.05) among experimental groups in frozen-thawed samples are represented with different capitals superscripts letters (A, B). Significant differences (*p* < 0.05, *p* < 0.001, *p* < 0.0001) between cooling and frozen-thawed samples in the same experimental group (sexual regime) are represented with asterisks (*, ***, ****, respectively). Significant differences (*p* < 0.05) among the differential rate of the experimental groups are represented with different lowercase superscripts letters (a, b). The same twenty-one males were analyzed in each experimental group.

In frozen-thawed samples, TM from 10 CW decreased significantly (*p* < 0.05) compared to 4 CW (Figure 1A). In contrast, 0 CW did not show significant differences to 4 CW and 10 CW (Figure 1A). However, in cooled samples, TM did not show significant differences among each experimental group (0 CW, 4 CW, 10 CW) (Figure 1A). As expected, TM decreased significantly (*p* < 0.05) in frozen-thawed samples in comparison with cooled samples, within each experimental group (0 CW, 4 CW, 10 CW) (Figure 1A).

Concerning PM, in frozen-thawed samples, it decreased significantly (*p* < 0.05) in 10 CW and 0 CW in comparison with 4 CW (Figure 1B). Nevertheless, in cooled samples, it did not show significant differences among each experimental group (0 CW, 4 CW, 10 CW) (Figure 1B). Moreover, PM decreased significantly (*p* < 0.05) in frozen-thawed samples in comparison with cooled samples, in the three experimental groups (0 CW, 4 CW, 10 CW) (Figure 1B).

With respect to FPM, in frozen-thawed samples, it decreased significantly (*p* < 0.05) in 10 CW and 0 CW in comparison with 4 CW (Figure 1C). In the same way, in cooled samples, FPM decreased significantly (*p* < 0.05) in 10 CW in comparison with 0 CW (Figure 1C). Nevertheless, 4 CW did not show significant differences to 0 CW and 10 CW (Figure 1C). As expected, FPM decreased significantly (*p* < 0.05) in frozen-thawed samples in comparison with cooled samples, within each experimental group (0 CW, 4 CW, 10 CW) (Figure 1C).

Concerning differential rate between cooled and cryopreserved samples, the DR of TM did not show significant differences among each experimental group (Figure 1D). In contrast, the DR of PM and FPM decreased significantly (*p* < 0.05) in 0 CW and 10 CW in comparison to 4 CW (Figure 1E,F).

**Figure 2:**
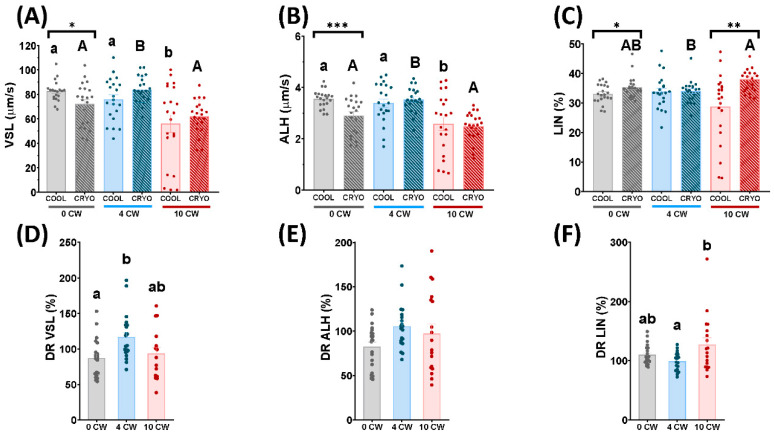
Ram sperm kinetics parameters in cooling (COOL) and frozen-thawed (CRYO) samples and differential rate (cooling/freezing) depending on the sperm collection frequency: without sperm collection (0 CW), 4 sperm collections per week (4 CW), 10 sperm collections per week (10 CW). (**A**) Velocity according to the straight path (VSL, μm/s); (**B**) Amplitude of the lateral displacement of the sperm head (ALH, μm); (**C**) Linearity (LIN, %); (**D**) Differential rate of velocity according to the straight path (VSL, %); (**E**) Differential rate of amplitude of the lateral displacement of the sperm head (ALH, %); (**F**) Differential rate of linearity (LIN, %). Significant differences (*p* < 0.05) among experimental groups in cooling samples are represented with different lowercase superscripts letters (a, b). Significant differences (*p* < 0.05) among experimental groups in frozen-thawed samples are represented with different capitals superscripts letters (A, B). Significant differences (*p* < 0.05, *p* < 0.01, *p* < 0.001) between cooling and frozen-thawed samples in the same experimental group are represented with asterisks (*, **, ***, respectively). Significant differences (*p* < 0.05) among the differential rate of the experimental groups are represented with different lowercase superscripts letters (a, b). The same twenty-one males were analyzed in each experimental group.

In frozen-thawed samples of 0 CW and 10 CW, the pattern of sperm movement concerning VSL decreased significantly (*p* < 0.05) in comparison with 4 CW (Figure 2A). Similarly, in cooled samples, VSL decreased significantly (*p* < 0.05) in 10 CW in comparison with 4 CW and 0 CW (Figure 2A). Nevertheless, this pattern of sperm movement did not show significant differences between 0 CW and 10 CW (Figure 2A). Moreover, VSL decreased significantly (*p* < 0.05) in cooled samples compared to frozen-thawed samples in 0 CW (Figure 2A).

Concerning ALH, in frozen-thawed samples this kinetic parameter decreased significantly (*p* < 0.05) in 0 CW and 10 CW, in comparison with 4 CW (Figure 2B). In the same way, in cooled samples, it decreased significantly (*p* < 0.05) in 10 CW in comparison with 4 CW and 0 CW (Figure 2B). Nevertheless, this pattern of sperm movement did not show significant differences between 0 CW and 10 CW (Figure 2B). In addition, ALH decreased significantly (*p* < 0.05) in cooled samples compared to frozen-thawed samples in 0 CW (Figure 2B).

Moreover, in frozen-thawed samples, the LIN pattern suffered a significant increase (*p* < 0.05) in 10 CW compared to 4 CW (Figure 2C). Nevertheless, 0 CW did not show significant differences to 4 CW and 10 CW (Figure 2C). On the other hand, LIN did not show significant differences among each experimental group in cooled samples (Figure 2C). Comparing cooled and frozen-thawed samples, LIN increased significantly (*p* < 0.05) in frozen-thawed samples in 0 CW and 10 CW (Figure 2C).

Concerning differential rate, DR of VSL decreased significantly (*p* < 0.05) in 0 CW in comparison with 4 CW (Figure 2D). Nevertheless, 10 CW did not show significant differences to 0 CW and 4 CW concerning to VSL (Figure 2D). ALH did not show significant differences among each sperm collection regime (Figure 2E). Moreover, the pattern of sperm movement concerning LIN increased significantly (*p* < 0.05) in 10 CW compared to 4 CW (Figure 2F). Although, 0 CW did not show significant differences in comparison to 4 CW and 10 CW in this parameter (Figure 2F).

### 3.2. Flow Cytometry Analyses

**Figure 3:**
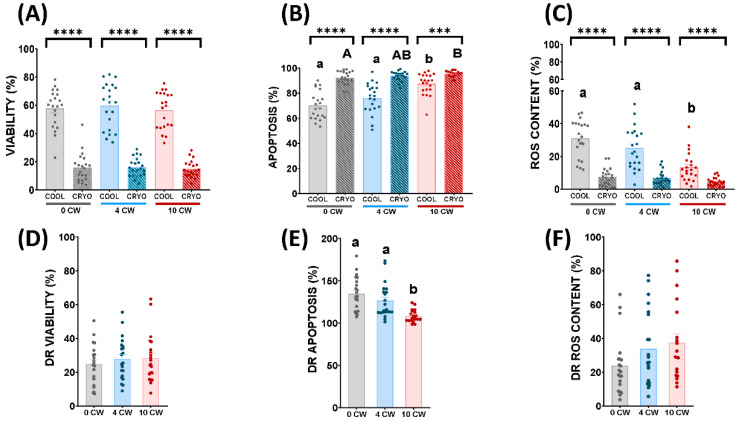
Ram sperm multiparametric analyses by flow cytometry in cooling (COOL) and frozen-thawed (CRYO) samples and differential rate (cooling/freezing) depending on the sperm collection frequency: without sperm collection (0CW), 4 sperm collections per week (4 CW), 10 sperm collections per week (10 CW). (**A**) Viability (%) (Zombie negative cells); (**B**) Apoptosis (%) (Caspase 3/7 positive cells); (**C**) ROS levels (%) (CellROX-positive cells); (**D**) Differential rate of viability (%); (**E**) Differential rate of apoptosis (%); (**F**) Differential rate of ROS content (%). Significant differences (*p* < 0.05) among experimental groups in cooling samples are represented with different lowercase superscripts letters (a, b). Significant differences (*p* < 0.05) among experimental groups in frozen-thawed samples are represented with different capitals superscripts letters (A, B). Significant differences (*p* < 0.001, *p* < 0.0001) between cooling and frozen-thawed samples in the same experimental group are represented with asterisks (***, ****, respectively). Significant differences (*p* < 0.05) among the differential rate of the experimental groups are represented with different lowercase superscripts letters (a, b). The same twenty-one males were analyzed in each experimental group.

Viability did not show any significant differences (*p* < 0.05) among each collection regime in cooled and frozen-thawed samples (Figure 3A). As expected, viability decreased significantly (*p* < 0.05) in frozen-thawed samples compared to cooled samples in the three experimental regimes (Figure 3A).

On the other hand, apoptosis significantly increased (*p* < 0.05) during 10 CW in respect to 0 CW in frozen-thawed samples (Figure 3B). In contrast, samples of 4 CW did not show significant differences between 0 CW and 10 CW, respectively (Figure 3B). In cooled samples, it increased significantly (*p* < 0.05) in 10 CW in comparison to 0 CW and 4 CW (Figure 3B). Nevertheless, 0 CW did not show significant differences with 4 CW in this parameter (Figure 3B). As might be expected, apoptosis increased significantly (*p* < 0.05) in frozen-thawed samples compared to cooled samples in the three experimental regimes (0 CW, 4 CW, 10 CW) (Figure 3B).

Finally, ROS content did not show any significant differences (*p* > 0.05) among experimental groups in frozen-thawed samples (Figure 3C). However, in cooled samples, it decreased significantly (*p* < 0.05) in 10 CW in comparison to 0 CW and 4 CW (Figure 3C). Although, 0 CW did not show significant differences to 4 CW (Figure 3C). Comparing cooled and frozen-thawed samples, ROS content decreased significantly (*p* < 0.05) in frozen-thawed samples in the three experimental regimes (Figure 3C).

Concerning differential rate, the DR of viability did not show any significant differences among each experimental group (Figure 3D). Nevertheless, apoptosis decreased significantly (*p* < 0.05) in 10 CW in comparison with 0 CW and 4 CW. (Figure 3E). Samples of 4 CW did not show any significant differences compared to 0 CW in apoptosis (Figure 3E). In addition, ROS content did not show any significant differences among each sperm collection regime (Figure 3F).

### 3.3. Redox Analyses: Oxidation-Reduction Potential, SOD and GPX Activity

**Figure 4:**
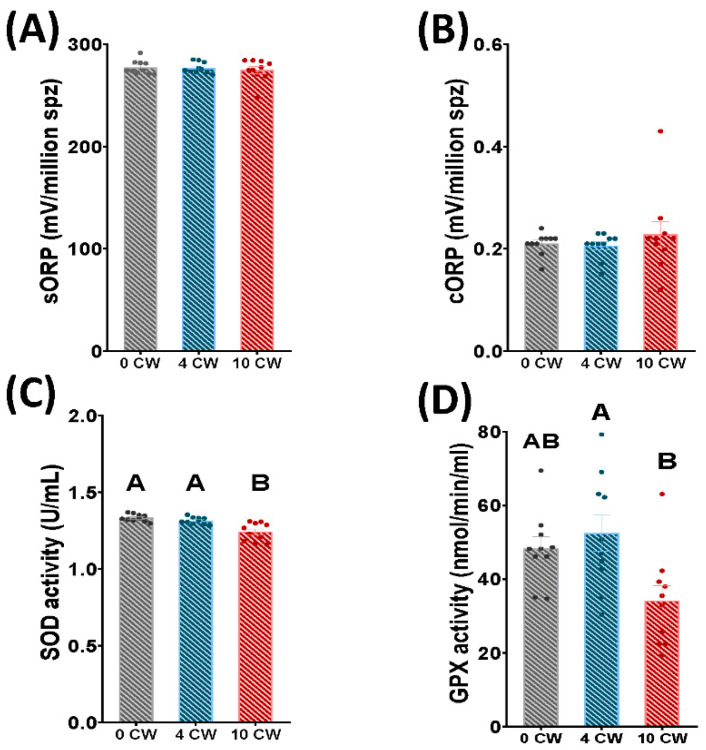
Ram sperm MiOXSYS^®^ indexes and, GPX and SOD enzymatic activity in frozen-thawed samples depending on the sperm collection frequency: without sperm collection (0 CW), 4 sperm collections per week (4 CW), 10 sperm collections per week (10 CW). (**A**) Integrated balance of oxidants and reductants (sORP, μC/10^6^ sperm); (**B**) Capacitance ORP (cORP, mV/10^6^ sperm); (**C**) Superoxide dismutase (SOD) activity (U/mL); (**D**) Glutathione peroxidase (GPX) activity (nmol/min(mL). Significant differences (*p* < 0.05) among experimental groups in frozen-thawed samples are represented with different letters. The same ten males were analyzed in each experimental group.

The integrated balance of oxidants and reductants (sORP) and capacitance ORP (cORP) did not show differences among each experimental condition in cryopreserved samples (without sperm collection per week, 4 sperm collections per week, 10 sperm collections per week) (Figure 4A,B).

Concerning enzymatic activities, SOD and GPX activity significantly decreased (*p* < 0.05) in 10 CW compared to 0 CW and 4 CW (Figure 4C,D). In contrast, in 0 CW no significant differences were found in SOD and GPX enzymatic activity compared to 4 CW (Figure 4C,D).

**Figure 5:**
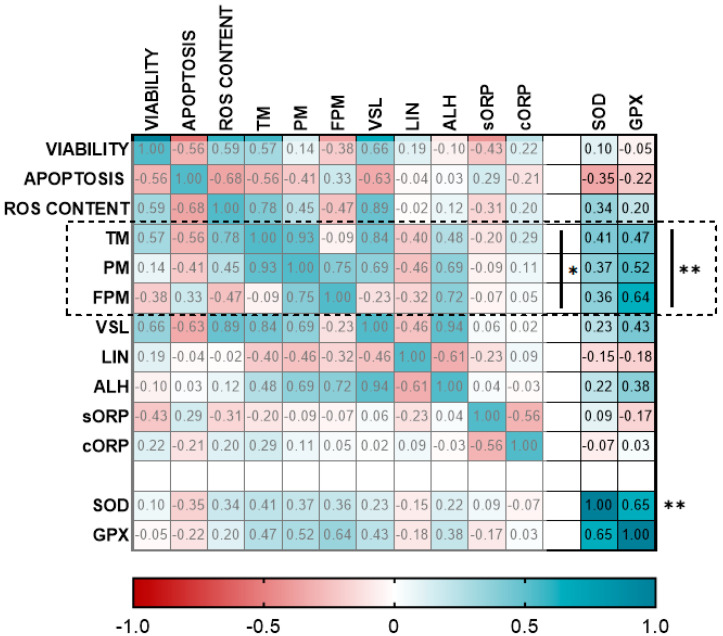
Correlation matrix of all sperm quality markers evaluated, highlighting the correlations of SOD and GPX activities. The three experimental groups: without sperm collection (0 CW), 4 sperm collections per week (4 CW), 10 sperm collections per week (10 CW) were included to calculate the correlation matrix. The R squared value between two parameters is represented in each cell. Blue indicates positive correlations and red indicates negative correlations. The color intensity represents the strength of the correlation between two sperm quality parameters. A total of ten males were analyzed (the same number of samples is required in this analysis). Asterisks show significant correlations among sperm quality parameters. The number of asterisk (*) indicates the significance levels: (*) indicates *p* < 0.05 and two asterisks (**) indicates *p* < 0.01.

Different correlations among SOD and GPX activities were found when we analyzed the correlations of each enzyme with motility parameters (Figure 5). SOD and GPX activity levels presented the highest correlation (R2 = 0.65) (*p* < 0.01) (Figure 5). Concerning motility parameters, TM, PM and FPM presented a significant and positive correlation with GPX activity (*p* < 0.01) and SOD activity (*p* < 0.05) (Figure 5).

## 4. Discussion

Cryopreservation is the most efficient method for long-term semen preservation used in mammalian species [5,41]. Until now, the improvement of thawed semen quality in ram sperm has been carried out by optimization of traditional freezing protocols, considering the addition of different substances [31] (cryoprotective agents [32], extender mediums [42] or antioxidants [30,43]), or testing different freezing and thawing rates [32,33].

Our study planned to attend to the influence of semen collection frequency on sperm quality after thawing, compared to sperm quality before freezing. Previous works have demonstrated a detrimental effect after an intensive frequency of semen collection in fresh samples in ram sperm [36,38]. This aspect has not been considered as a determinant factor related to frozen-thawed sperm quality in sperm dose elaboration in ovine species until now. Nevertheless, in insemination centers the work protocols of semen collection frequency are determined by dose demand, always within the capabilities of the males and never allowing for male exhaustion. Usually, high genetic trait males are the most demanded by insemination centers. Therefore, the possible negative impact of 10 CW could decrease the performance of insemination programs for the most valuable ram males.

In this work, we propose an alternative strategy for the improvement of thawed sperm quality through the selection of the optimal time to carry out the semen collection, considering work protocols used in reproduction centers, based on the demand of semen doses. Consequently, we performed multiparametric analyses of ram sperm quality at different levels, combining different traditional analyses with new ones, in cooled and frozen-thawed samples obtained in three experimental groups submitted at different frequencies of semen collection: without semen collection (0 CW), four collections per week (4 CW), and ten collections per week (10 CW) for a month. Moreover, we focused our study on redox balance analyses employing novel and accuracy technologies, such as MiOXSYS^®^, SOD and GPX activity, to study the existence of different cryo-resistance among sperm collection regimes, due to our interest in improving frozen-thawed sperm quality.

Attending to motility and kinetics parameters carried out by CASA, in accordance with previous studies [44], we observed that cooled samples did not show any significant differences in TM and PM among each semen collection regime (Figure 1A,B). However, FPM in these samples decreased significantly (*p* < 0.05) in 10 CW in comparison to 0 CW (Figure 1C). Frozen-thawed samples of males submitted to 0 CW and 10 CW, on the other hand, exhibited a significant (*p* < 0.05) detrimental effect in both PM and FPM (Figure 1B,C). In the case of TM, thawed samples presented a significant (*p* < 0.05) decrease in 10 CW compared to 4 CW (Figure 1A). These results could be explained as a consequence of a drastic temperature change caused by the cryopreservation process. Although non-significant differences were detected regarding TM and PM in 10 CW, compared to 4 CW, in cooled samples, some sublethal damage in sperm that we did not detect was occurring, making these experimental groups more susceptible to cryopreservation. Probably, 0 CW and 10 CW could provoke more severe damage in sperm samples than 4 CW in the cryopreservation process. Despite the increase in motility parameters observed before freezing, linearity showed a significant decrease (*p* < 0.05) with respect to frozen-thawed samples in 0 CW and 10 CW. This fact was previously described by Martinez-Pastor et al. [45] in an analysis of subpopulations of red deer regarding sperm velocity (slow and nonlinear, fast and linear, and fast and nonlinear) during the cryopreservation process. Moreover, some alterations in the patterns of sperm movement were observed, depending on sperm collection frequency in frozen-thawed samples (Figure 2A–C). This fact could be explained as a consequence of freezing injury, which could make the damage associated with an intensive regimen more severe. Also, it has been described that after intensive sexual regime kinetics parameters, such as VSL, ALH, BCF or STR, suffered a detrimental effect in dromedary camels [46]. However, until now, this is the first time that the influence of semen collection frequency on post-thawed sperm quality in rams has been studied. Multiparametric flow cytometry analyses revealed some sperm quality parameters were affected by the three different sperm collection regimes. We did not find significant differences in cooled and thawed samples concerning viability (Figure 3A), despite previous studies describing low sperm viability after high semen collection frequency in rams [38] and llamas [47]. Nevertheless, when a deeper assessment was carried out, cooled and frozen-thawed samples of ram semen under a 10 CW regime showed a significant increase (*p* < 0.05) in apoptosis levels, increasing the activation of caspase 3 and 7, in comparison to 0 CW and 4 CW (Figure 3B, Appendix A). Apoptosis increase is a consequence of cellular damage caused by lipid peroxidation, and loss of membrane integrity is associated with an increase of ROS content in sperm cells [48]. Despite apoptosis levels being higher in thawed samples, in comparison with cooled samples, the DR of apoptosis revealed a lower value in 10 CW in comparison with 4 CW and 0 CW, due to great differences found in this parameter in cooled samples (Figure 3B). This could be explained as a consequence of sexual regime influence being greater than the cryopreservation damage. It is important to remark, our study was carried out over 3 months during the ovine reproductive season with an increasing collection frequency (0 CW, 4 CW, 10 CW); therefore, an increase in seminal quality could be expected [49]. Nonetheless, we observed a decrease in the sperm quality of thawed samples submitted to 10 CW. This fact confirmed the importance of sexual regime on sperm quality that could be underestimated due to the effect of breeding season progression.

Unexpectedly, concerning to ROS content detected by CellROX, a significant decrease (*p* < 0.05) in cooled samples in 10 CW, in comparison with 0 CW and 4 CW (Figure 3C), was evidenced. This could be explained by previous works showing that blocking electron transfer provoked irruption in superoxide anion production, which is the target molecule of the CellROX probe, according to the manufacturer´s protocol [50]. For this reason, an increase in superoxide anion (positive CellROX cells) was associated with high mitochondrial activity rather than oxidative stress. Previously Desai et al. [51] did not find significant correlation between ROS content levels, sperm motility and sexual regime in humans, considering ROS level variations associated with physiologic, seasonal or environmental causes. However, we did not find any significant differences in frozen-thawed samples among the experimental groups subjected to different sexual regimes (Figure 3C). The DR of ROS content did not show any significant differences among each experimental group. In this case, the cryopreservation process and its oxidative effects could mask the damage caused by a period of 10 sperm collections per week. For this reason, a deeper assessment was carried out to study the presence of different cryoresistances among experimental groups. More accuracy methods were employed based on new technologies (MioXSYS^®^), and specific enzymatic activities related to redox balance (such as GPX and SOD) in frozen-thawed samples.

In the last decade, novel technologies (MiOXSYS^®^) have been developed as a measure of redox balance in a global way, and as precision oxidation-reduction potential (ORP) markers [52]. These novel technologies have been successfully applied after cryopreservation in different species, such as rams [53], stallions [54] and humans [26]. Nevertheless, in our study MiOXSYS^®^ did not reveal any differences in sperm quality among experimental conditions (Figure 2D,E). This fact could be explained by the sensibility of MiOXSYS^®^. In our previous studies, MiOXSYS^®^ technology has usually been employed to compare ram sperm with a highly different level of damage and oxidative stress: fresh, cooled and thawed samples that trigger a redox imbalance reflected in MiOXSYS^®^ indexes [53,55]. In this study, all samples were cryopreserved, and the great cryopreservation effect on redox balance could mask the differences among our experimental conditions (different frequency of semen collection). For this reason, we decided to employ more specific techniques to determine redox status by measuring specific antioxidant enzymes. SOD and GPX enzyme activities, in seminal plasma samples. Seminal plasma contains different enzymatic antioxidants (SOD, GPX, CAT) and non-enzymatic antioxidant levels to counteract excessive ROS. There is some evidence that existing predominant antioxidant enzymes related to frozen-thawed quality depend on mammalian species: SOD in stallions [25], rams [17] and bulls [56]; CAT in donkeys [57]. Moreover some differences were described regarding antioxidant capacity of seminal plasma regarding animal species (higher in donkeys in comparison with stallions) [58]. Also antioxidant enzyme quantity depends on each individual but no intra-individual differences exist (different ejaculates) [56]. Furthermore, previous studies in stallions detected a higher SOD and GPX activity in seminal plasma than in sperm and reproductive tissue, being seminal plasma the best sample to quantify these antioxidant responses [59].

In our study, SOD and GPX enzyme activities of seminal plasma, revealed a decrease in sperm samples of ram males subjected to 10 CW, in comparison to 4 CW and 0 CW (Figure 2F and Figure 5). We found that SOD and GPX enzyme activities were positivity correlated between them (R2 = 0.65) (Figure 5). In addition, previous studies described this correlation between both enzymes in several species, such as rams [23], stallions [25] or humans [60]. Furthermore, some correlations were found in our work with motility parameters: positive correlation was found among SOD and GPX activity with TM, PM and FPM (Figure 5). Significant correlation between conventional parameters were not shown in our work because previously published works demonstrated the existence of these correlations [30,53]. In accordance with our results, previous studies demonstrated correlation of enzymatic activity with motility parameters in rams [23], stallions [25,61], boars [62], buffalos [61] and donkeys [58]. Nevertheless, we found significant differences in motility and kinetics parameters between 0 CW and 4 CW that were not reflected in SOD and GPX levels. Confirming our results, a significant decrease in total and progressive motility was previously demonstrated in an abstinence period in comparison to regular ejaculation frequency in humans [35,63]. This decrease could be explained as a result of sperm storage in cauda epididymis where they were exposed to sperm motility quiescence factors that could have a negative impact on sperm motility after ejaculation [64,65]. These quiescence factors included a decrease in pH as a consequence of carbon dioxide produced by the storage of sperm in the epididymis causing an increase in hydrogen ion levels, or a high potassium to sodium ratio in epididymal fluid [65]. In addition, some authors did not demonstrate significant correlation between ROS content levels, sperm motility and sexual regime in humans [51]. These results demonstrated that SOD and GPX activity are positively involved in sperm cryotolerance, being an efficient protection against oxidative stress. Also, it has been demonstrated that SOD and GPX activity levels in seminal plasma were not sufficient to protect frozen sperm from oxidative stress after a monthly period of 10 semen collections per week. This oxidative stress could be the cause of the observed alterations in sperm quality in frozen-thawed samples: a detrimental effect in motility and kinetics parameters, and a high apoptosis level.

## 5. Conclusions

This work allows us to establish an alternative factor to be studied regarding sperm dose elaboration to improve thawed sperm quality: semen collection frequency. In this study we observed the relationship between higher semen collection frequency with lower frozen-thawed quality. Specifically, we found alterations in sperm motility, kinetic parameters and high apoptosis levels after a monthly period of 10 sperm collections per week. Although we observed changes in the activity of antioxidant enzymes, the role of antioxidant enzymes to counteract oxidative stress could not be enough to protect sperm from cryopreservation after the monthly period of 10 sperm collections per week. This innovative approach could be applied to improve freezing protocols, guaranteeing a high seminal quality in reproduction centers.

## Figures and Tables

**Figure A1:**
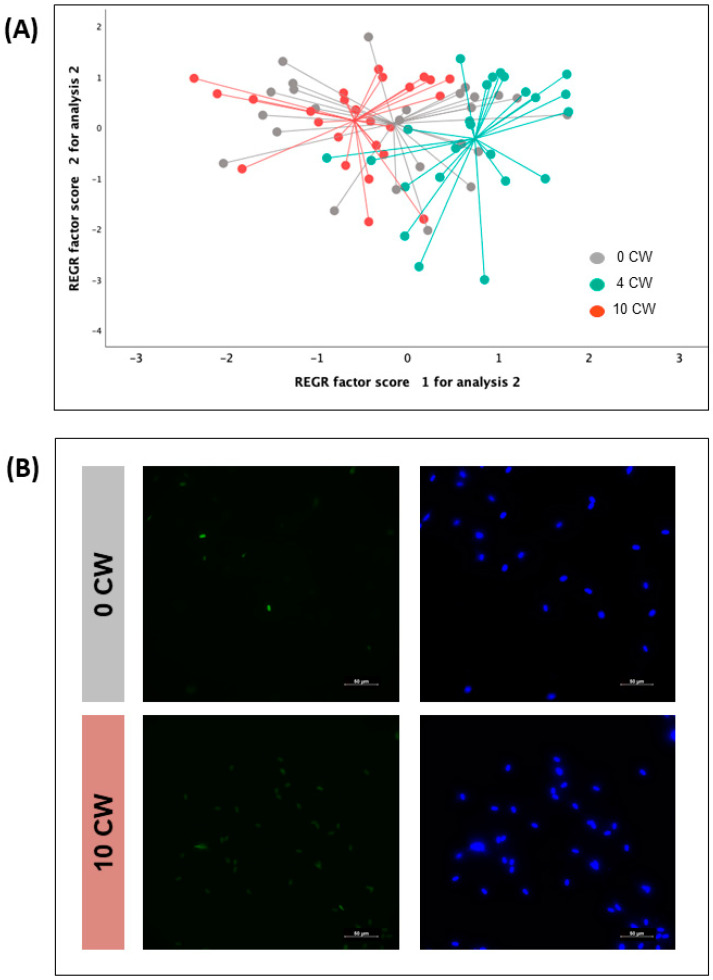
(**A**) Representation of twenty-five rams in frozen-thawed samples: scatter plot of males depending on the sperm collection frequency (zero sperm collection per week (0 CW), 4 collections per week (4 CW), 10 collections per week (10 CW) separated by the two principal components obtained by principal component analysis (PCA) to reduce the dimensionality of the dataset. (**B**) Ram sperm confocal images of frozen-thawed samples: apoptosis marker (CellEvent Caspase-3/7 Green) and nuclear marker (Hoechst) in zero sperm collection per week (0 CW) and 10 sperm collections per week (10 CW).

## Data Availability

The data presented in this study are available on request from the corresponding author.
